# Bleb Morphology Using Anterior-Segment Optical Coherence Tomography after Ahmed Glaucoma Valve Surgery with Tenon Capsule Resection

**DOI:** 10.1155/2020/8386135

**Published:** 2020-10-22

**Authors:** Faried M. Wagdy

**Affiliations:** Ophthalmology Department, Faculty of Medicine, Menofia University, Shebeen El-Kom, Egypt

## Abstract

**Objectives:**

To evaluate the internal morphology of blebs using anterior-segment optical coherence tomography (AS-OCT) and the surgical outcomes of Ahmed glaucoma valve (AGV) surgery with Tenon capsule resection compared to conventional AGV surgery in patients with refractory glaucoma. *Patients and Methods*. This randomised prospective study included 30 eyes from 30 patients (age range: 42–55 y) with refractory glaucoma from March 2018 to February 2020. The study included two groups: AGV with the Tenon capsule resection group (*n* = 15) and the conventional AGV surgery group (*n* = 15). Follow-up continued until 6 months after surgery. The primary outcome was intraocular pressure (IOP) and its association with the number of postoperative glaucoma medications, best corrected visual acuity, visual field, and postoperative complications. The internal morphology of the blebs in both groups was evaluated at 1-day, 1-week, 1-month, 3-month, and 6-month follow-up using AS-OCT in terms of the consequent changes in bleb wall thickness, bleb wall reflectivity, and bleb cavity morphology throughout the 6-month follow-up period.

**Results:**

A significant reduction in IOP was found in both groups, with a greater reduction in group I, where the mean IOP decreased from 32.73 ± 2.12 mmHg in the preoperative period to 13.33 ± 1.59 mmHg after 6 months, whereas in group II, the mean IOP decreased from 33.2 ± 2.21 mmHg in the preoperative period to 14.27 ± 1.44 mmHg after 6 months (*p* value <0.05). The difference between the 2 groups in terms of the decrease in IOP was insignificant except at 1 and 3 months, where there was a significant difference (*p* value = 0.016 and 0.01 at 1 and 3 months, respectively). The bleb analysis revealed a significant reduction in the wall thickness in both groups at 1 and 3 months, which was mostly associated with the hypertensive phase. In group I, the bleb wall thickness decreased from 754.67 ± 53.93 *μ*m in the first postoperative day to 684 ± 81.66 *μ*m and 671.6 ± 69.48 *μ*m at 1 and 3 months, respectively, while in group II, the bleb wall thickness decreased from 707.13 ± 31.7 *μ*m in the first postoperative day to 499.53 ± 99.1 *μ*m and 506 ± 76.91 *μ*m at 1 and 3 months, respectively. There was a significant reduction in AS‐OCT, and bleb reflectivity was insignificant throughout the follow-up period (*p* value >0.05). Regarding postoperative complications, the hypertensive phase occurred more frequently in group II (6 eyes, 40%) than in group I (2 eyes, 13.3%). Other complications were also reported more frequently in group II such as hypotony, shallow anterior chamber (AC), and tube exposure.

**Conclusion:**

AS-OCT was beneficial in the analysis of bleb morphology after AGV surgery where there were more diffuse functioning multicystic blebs and less thinning in the bleb wall thickness during the hypertensive phase after resection of the Tenon capsule, which might be related to the less incidence of fibrosis around the surgical site.

## 1. Introduction

Glaucoma drainage devices are important therapeutic tools for the management of refractory glaucoma with high success rates [1]. Ahmed glaucoma valve (AGV) is a glaucoma drainage shunt that is performed in refractory glaucoma, especially after the failure of previous conventional surgeries. It allows the aqueous to flow directly through the valve tube and lowers the complications related to this technique [2–4]. Following AGV surgery, the bleb is subjected to two stages. The early hypotensive phase is followed by a hypertensive phase that tends to often occur at 1 month, and often stabilises at 6 months after the AGV implantation. The cause is still poorly understood [5]. The hypertensive phase occurred in 82% of the cases after Ahmed valve implantation. Partial intraoperative Tenon capsule resection using the Mitomycin C adjunctive may be effective in developing thin avascular blebs if performed during the Ahmed glaucoma valve surgery [6].

A novel technique of Tenon capsule resection combined with an AGV implantation was developed to clarify the effectiveness and the safety of the Tenon capsule resection, when it is performed during an AGV surgery, bringing a relatively low incidence of tube exposure and the hypertensive phase [7].

AS-OCT is a noncontact diagnostic method that presents a cross-sectional, three-dimensional, and high-resolution image of the anterior segment of the eye, with an axial resolution that ranges from 3 to 20 *μ*m. There are two AS-OCT devices that are currently available: Visante-OCT (Carl Zeiss Meditec; USA) and slit-lamp OCT (SL-OCT; Heidelberg Engineering GmbH, Germany) [8, 9]. AS-OCT provides qualitative and quantitative assessments of the most important structures related to AGV surgery and can be used to assess the position of the tube in the anterior chamber (AC) and to determine the bleb characteristics and function. Although bleb imaging is usually performed by ultrasound biomicroscopy (UBM) [10, 11], AS-OCT can be useful to assess the bleb morphology and function and to differentiate between functioning and nonfunctioning blebs [12]. AS-OCT also can measure the maximum bleb wall thickness and the maximum bleb wall reflectivity after AGV surgery, which are usually affected by subsequent fibrosis in the bleb after surgery and are therefore used as indicators for the success of the surgery [13].

The present study was aimed to evaluate the internal bleb morphology of blebs using AS-OCT and the surgical outcomes of Ahmed glaucoma valve (AGV) surgery with Tenon capsule resection as compared to conventional AGV surgery in patients with refractory glaucoma.

## 2. Patients and Methods

Thirty eyes with refractory glaucoma, in spite of the maximally tolerated medication after previous trabeculectomy surgery, were included in a prospective, randomised, controlled clinical study that used a computer-generated random number table. This study was conducted in Menofia University Hospital between March 2018 and February 2020. Institutional research board committee approval was obtained. Two groups were studied: group I for the AGV surgery with Tenon capsule resection (*n* = 15 eyes) and group II for the conventional AGV surgery (*n* = 15 eyes). Preoperatively, all the patients were subjected to IOP measurement using the Goldman applanation tonometer, visual acuity assessment (VA) using Snellen E chart, visual field analysis (VF) by Humphrey visual field analyser, Angle examination by goniolens, optic disc examination by Volk+90 lens, slit lamp examination for the assessment of the corneal clarity, and any corneal touch with the tube and anterior chamber depth. All the patients had peribulbar anaesthesia by the same surgeon.

## 3. Surgical Technique

### 3.1. Operative Technique in Group II (Conventional AGV Surgery)

The AGV device should be primed before the surgery. Priming is performed using a 26G cannula, injecting 1 cc of balanced salt solution (BSS). The superior-temporal conjunctiva was incised and appropriately cauterised. Mitomycin C (MMC) at a concentration of 0.2 mg/ml was applied using 3 sponges soaked with MMC that were placed over the sclera and left for 2 minutes, followed by irrigation using a balanced salt solution, and the valve body plate was placed approximately 10 mm posterior to the corneal limbus and fixed to the underlying sclera with Nylon 10-0 sutures. A two-third thickness longitudinal scleral flap (2 mm × 6 mm) was created, and the tube was then inserted for 2-3 mm into the AC parallel to the iris plane. The flap was closed tightly with Nylon sutures to avoid leakage around the tube. The tube was ligated to the underlying sclera with an 8-0 Vicryl. Conjunctiva and Tenon capsule were sutured with Nylon 10-0.

#### 3.1.1. Operative Technique in Group I (AGV Surgery with Tenon Capsule Resection)

The same steps were performed for the conventional AGV surgery, the difference being that the Tenon capsule was dissected from the limbus up to the posterior border of the AGV body plate. Finally, the conjunctiva was closed with nylon sutures.

#### 3.1.2. Postoperative Management

Postoperative treatment included combined antibiotic and steroid eye drops every four hours in the first week with a gradual tapering after 2 weeks.

The surgical outcomes were evaluated based on the following:

(i) IOP and the number of glaucoma medications: a complete success was defined as an IOP lower than 21 mmHg without treatment, a qualified success was defined as an IOP lower than 21 mmHg with medical treatment, failure was defined as an IOP more than 21 mmHg during any time throughout the follow-up period with medical treatment. The hypertensive phase was defined as an elevated IOP more than 21 mmHg during the first 3 months after surgery. If the hypertensive phase could not be lowered with medications, it would be considered a failure. Patients were divided into two categories: the hypertensive and the nonhypertensive categories. Hypotony was defined as an IOP <6 mmHg. The number of postoperative antiglaucomatous medications was identified.
(ii) Visual acuity: visual acuity assessment (VA) using the Snellen E chart was performed. The final postoperative BCVA changes were classified as “worsened”, “stable”, or “improved” when compared to the preoperative BCVA. A change of one line of Snellen visual acuity or less was defined as stable, whereas greater changes were defined as worsened or improved accordingly.
(iii) Visual field: visual field assessment was based on Brusini Classification based on Mean Deviation (MD) and Corrected Pattern Standard Deviation (CPSD) Values:
    Stage 0: both MD and CPSD within normal limits.
    Stage 1: MD between −3 and −5 dB and CPSD ≤3 dB, or MD <−3 dB and CPSD between 3 and 5 dB, or both MD and CPSD between −3 and −5 dB.
    Stage 2: MD >−5 and <−8 dB and CPSD <−8 dB , or MD <−3 dB and CPSD >5  and <8 dB.
    Stage 3: MD between −8 and −12 dB, or CPSD ≥8 dB.
    Stage 4: MD ≥−12 dB and <−20 dB.
    Stage 5: MD ≥−20 dB [14].
(iv) Postoperative complications: all intraoperative and postoperative complications were recorded.
(v) Anterior segment OCT assessment: a Spectralis OCT (Heidelberg Engineering, Heidelberg, Germany) was performed to visualise the postoperative blebs at 1 week, 1 month, 3 month, and 6 months. AS-OCT was performed in a room with a constant and minimal degree of room illumination. Patients were initially asked to fixate their eyes at an inferonasal position to facilitate the imaging of the superotemporal bleb and the nearby angle and AC. Automatic real-time of eight frames and 41 sections with a 139 *μ*m interval were used for imaging the cross section of the bleb. The maximal bleb wall thickness was calculated by measuring in micrometres the maximal distance 3.5 mm away from the end point of the tube. Images with quality scores greater than 25 were included. The maximal bleb wall thickness was calculated by measuring in micrometres the maximal distance between the first reflective signal from the conjunctiva to the top of the sub-Tenon fluid space. The maximal reflectivity of the bleb wall was measured by exporting the scanned images as jpg files and then importing them into the ImageJ software program (Wayne Rasband, National Institutes of Health, Bethesda, MD, USA). Reflectivity was measured in the ellipses that the operator had marked on the bleb wall. The internal morphology of the blebs in both groups was evaluated using the anterior segment optical coherence tomography (AS-OCT) for consequent changes in bleb wall thickness, bleb wall reflectivity, and bleb shape throughout the 6 months of follow-up period.

In addition, optic disc examination using Volk + 90 lens and slit lamp examination, including bleb examination for its shape and vascularity, were performed throughout the 6 months of follow-up period.

### 3.2. Statistical Analysis

Results were statistically analysed by SPSS version 22 (SPSS Inc., Chicago, IL, USA). Nonpaired *t*-test was used for parametric data. Mann–Whitney and Friedman tests were used for nonparametric data. Chi-squared (*χ*^2^) and Fisher's exact tests were used for qualitative variables. Spearman test was used for detecting the strength and the direction of association between variables. A *p* value <0.05 was considered significant.

## 4. Results

**Figure 1:**
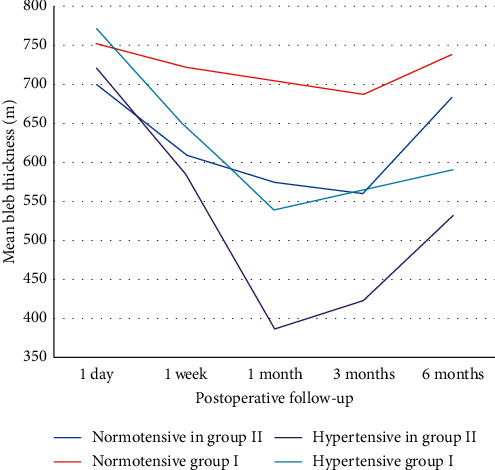
U-shaped pattern of the mean maximal bleb wall thickness.

**Figure 2:**
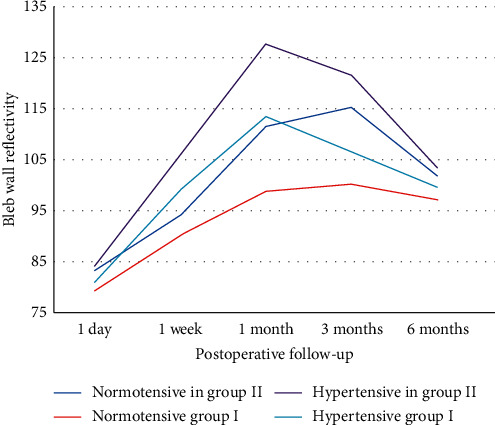
Inverted U-shaped pattern of the mean maximal bleb wall reflectivity.

**Figure 3:**
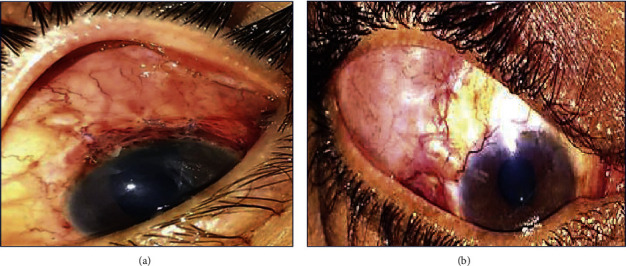
(a) A slit lamp biomicroscopy photo of a male patient in group I with IOP at 15 mmHg revealed a thin bleb with minimal vascularisation (3 months after surgery). (b). A slit lamp biomicroscopy photo of a female patient in group II with IOP 28 mmHg revealed a vascularised encysted bleb (1 month after surgery).

**Figure 4:**
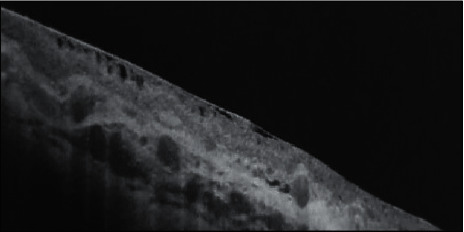
AS-OCT bleb imaging of a female patient in group I during the hypertensive phase at 3 months; IOP was 26 mmHg. AS-OCT revealed the presence of diffuse multicystic bleb cavity, diffuse hyporeflective conjunctiva with hydration, and intraconjunctival cysts. Maximal bleb wall thickness was 580 *μ*m, and bleb wall reflectivity was 103.

**Figure 5:**
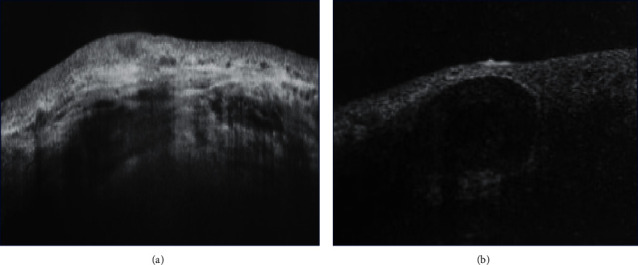
(a) AS-OCT bleb imaging of a female patient in group II at 1 month (hypertensive phase); IOP was 29 mmHg. AS-OCT revealed the presence of encapsulated bleb with dense hyperreflective bleb wall with monocystic fluid-filled bleb cavity; maximal bleb wall thickness was 375 *μ*m, and bleb wall reflectivity was 132. (b) AS-OCT bleb imaging of a male patient in group II at 3 months (hypertensive phase); IOP was 27 mmHg. AS-OCT revealed the presence of a few number of intraconjunctival cysts and minimal conjunctival hydration with dense hyperreflective areas within the bleb cavity (mostly due to fibrosis); the maximal bleb wall thickness was 389 *μ*m, and bleb wall reflectivity was 127.

**Figure 6:**
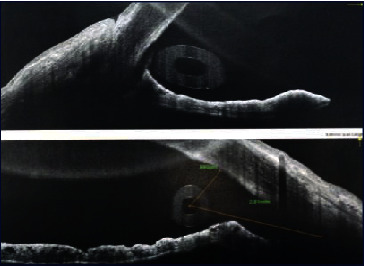
AS-OCT imaging 4 months after surgery of a male patient in group I IOP was 14 mmHg with representation of the tube opening in the AC, angle patency, and depth.

**Figure 7:**
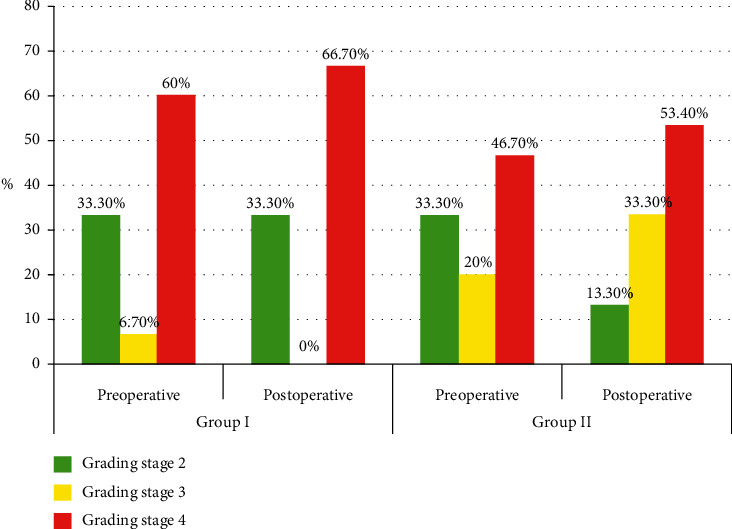
Comparison between the two groups with respect to the postoperative visual field.

The age of the patients ranged from 42 to 55 years, and there were 17 males and 13 females. In spite of the maximally tolerated medication after previous trabeculectomy surgery, the patients had a high intraocular pressure (IOP), with an IOP in the range of 30–36 mmHg (group I) and 30–37 mmHg (group II). These patients were diagnosed with three main types of refractory glaucoma; aphakic glaucoma (11 patients), pseudophakic glaucoma (15 patients), and pseudoexfoliation glaucoma (4 patients) (Table 1). This study showed a significant reduction in IOP in both groups throughout the follow-up period, with a greater reduction in group I, where the mean IOP decreased from 32.7 ± 2.1 mmHg in the preoperative period to 13.3 ± 1.6 mmHg after 6 months, whereas in group II, the mean IOP decreased from 33.2 ± 2.2 mmHg in the preoperative period to 14.3 ± 1.4 mmHg after 6 months (*p* value <0.05). The difference in IOP reduction between the 2 groups was insignificant except at 1 and 3 months, where there was a significant increase in the mean IOP in group II. The mean postoperative IOP in group II at 1 month and 3 months was 20.1 ± 6.4 mmHg and 17.0 ± 1.8 mmHg, respectively, while in group I, the mean postoperative IOP at 1 and 3 months was 14.9 ± 3.1 mmHg and 15.8 ± 3.4 mmHg, respectively (*p* = 0.016 at 1 month and 0.01 at 3 months) (Table 2). There were more patients with the hypertensive phase in group II (6 cases, 40%) when compared with group I (2 cases, 13.3%)) (Table 3). The bleb analysis revealed that there was a significant reduction in the maximal bleb wall thickness in both groups at 1 and 3 months, which was mostly associated with the hypertensive phase. In group I, the bleb wall thickness was 754.67 ± 53.93 *μ*m on the first postoperative day and decreased to 684 ± 81.66 *μ*m and 671.6 ± 69.48 *μ*m at 1 and 3 months, respectively, while in group II, the bleb wall thickness was 707.13 ± 31.7 *μ*m on the first postoperative day and decreased to 499.53 ± 99.1 *μ*m and 506 ± 76.91 *μ*m at 1 and 3 months, respectively (Table 4). AS‐OCT was also beneficial in describing the changes in maximal bleb wall reflectivity, which increased at 1 and 3 months and decreased again at 6 months, although the difference between both the groups with regard to the bleb reflectivity was insignificant throughout the follow-up period (Table 5). Maximal bleb wall thickness and maximal bleb wall reflectivity were analysed in those patients. There was a more significant reduction in the maximal bleb wall thickness at 1 and 3 months in group II. Maximal bleb wall thickness at 1 month was 540 ± 14.14 *μ*m in group I and was 387.83 ± 11.67 *μ*m in group II (*p* value <0.05). At 3 months, a mild increase occurred, where maximal bleb wall thickness was 567.5 ± 31.82 *μ*m in group I and 423.67 ± 9.31 *μ*m in group II (*p* value <0.05) (Table 6). There was a negative correlation between IOP and maximal bleb wall thickness in the eyes that presented with significant elevated IOP (Hypertensive phase) at 1 and 3 months in both groups. They also presented with a significant decrease in the maximal bleb wall thickness (Table 7). These measures increased again at 6 months and can be represented as a U-shaped pattern (Figure 1). The maximal bleb wall reflectivity increased at 1 and 3 months in both the groups. This increase was greater in group II, where the maximal bleb wall reflectivity was 127.66 ± 32.11 and 121.62 ± 18.23 at 1 and 3 months, respectively, while it was 113.48 ± 38.22 and 106.31 ± 19.56 in group I at 1 and 3 months, respectively. However, this difference between both the groups was insignificant (Table 5). This increase in the maximal bleb wall reflectivity subsided again at 6 months and can be represented as an inverted U-shaped pattern (Figure 2). A significant correlation was reported between the decrease in maximal bleb wall thickness and the increase in maximal bleb wall reflectivity at 1 and 3 months (Hypertensive phase) (Table 8). Postoperative slit lamp biomicroscopy for the studied groups clarified that externally, the blebs in group I were thinner and less vascular compared to those in group II. However, bleb morphology by AS-OCT provided more details, since the internal morphology of the blebs could be analysed (Figures 3(a) and 3(b)). All the blebs in group I were characterised by diffuse, fluid-filled, large multicystic cavities, numerous intraconjunctival cysts, and hyporeflective diffuse areas of conjunctival hydration that were seen in all the cases, except the 2 cases in the hypertensive phase. They were characterised by less fluid-filled multicystic cavities, intraconjunctival cysts, and conjunctival hydration (Figure 4) with a greater reduction in the maximal bleb wall thickness and an increase in the maximal bleb wall reflectivity (Table 6). Bleb shape in group II was different; here there was a uniform, fluid-filled cystic cavity without encapsulation in 9 cases (60%) that presented without the hypertensive phase, while 6 cases (40%) with the hypertensive phase were classified as a uniform cystic, fluid-filled cavity with encapsulation occurring in 5 cases (33.3) and a less diffuse multicystic bleb with dense fibrosis inside the bleb cavity in 1 case (6.7%) (Figure 5). Intraconjunctival cysts were very limited with less conjunctival hydration, which might be due to the onset of greater fibrous proliferation compared to group I. AS‐OCT was useful to determine the position of the AGV tube in the AC in addition to the patency of the AC angle (Figure 6). Regarding the postoperative complications, the hypertensive phase occurred more frequently in group II, where it occurred in 6 cases (40%) with a mean IOP of 25 mmHg (range, 21–29 mmHg), whereas it occurred in 2 cases at 1 month, with one case having an encapsulated bleb visible by AS-OCT; the 2 cases at 1 month were controlled 2 months later with two antiglaucomatous eye drops (Carbonic Anhydrase Inhibitor + Beta-blocker), while 4 cases with encapsulation (26.7%) needed to continue the antiglaucomatous eye drops till the 6-month follow-up. Three cases (20%) needed two antiglaucomatous eye drops (Carbonic Anhydrase Inhibitor + Beta-blocker) and 1 case (6.7%) received three antiglaucomatous eye drops (Carbonic Anhydrase Inhibitor + Beta-blocker + Prostaglandin Analogue). Only 2 cases (13.3%) in group I showed this hypertensive phase at 3 months, of which only 1 case (6.7%) with an IOP of 26 mmHg needed to continue with two antiglaucomatous eye drops (Carbonic Anhydrase Inhibitor + Beta-blocker). IOP was lowered to 15 mmHg while the other case presented with an IOP at 25 mmHg, which was lowered with two antiglaucomatous eye drops (Carbonic Anhydrase Inhibitor + Beta-blocker) after 2 weeks. This treatment was stopped 2 weeks later when the IOP was lowered to 8 mmHg; the IOP later increased back to 15 mmHg without any further treatment. More postoperative complications were reported in group II such as hypotony, shallow anterior chamber (AC), choroidal detachment, hyphaemia, and tube exposure (Table 3). The preoperative BCVA ranged between 2/60 (two meters) to 0.6 (by Snellen E-chart) in both the groups. With regard to the final postoperative BCVA in group I (*n* = 15), all cases remained stable (100%). For group II (*n* = 15), 4 had worsened (26.7%) and 11 remained stable (73.3%). In group II, 3 cases were associated with the hypertensive phase and showed a decline of 2 lines of the Snellen E-chart. The fourth case was complicated with choroidal detachment and showed a decline of 3 lines of the Snellen E-chart. In group I, the final postoperative visual field dropped from stage 3 to stage 4 in one case, while in group II, it dropped from stage 2 to stage 3 in 3 cases and from stage 3 to stage 4 in one case (Figure 7).

## 5. Discussion

Although bleb morphology can be evaluated using slit lamp biomicroscopy, it depicts only the external aspect of the bleb without assessing its internal structures [15–17] AS-OCT is helpful to obtain cross-sectional images of the internal structures of the bleb. In addition, AS-OCT has been shown to be useful in diagnosing Ahmed tube tip position and patency, even in patients with opaque corneas [18, 19].

This study has shown that there were more diffuse functioning multicystic blebs and less thinning of the bleb wall thickness during the hypertensive phase after resection of the Tenon capsule. This might be related to a lesser incidence of fibrosis and a reduced vascularity as well as a better hydration of the tissue around the surgical site (Figure 4). There was no encapsulation that might correlate to the resection of the Tenon, which could have reduced the fibrosis around the valve plate and might have explained the increase in the maximal bleb wall thickness as well as the reduction in the maximal bleb wall reflectivity observed in group I as compared to group II at 1 and 3 months. This could also explain the low incidence of cases with the hypertensive phase in this group.

Some studies reported that the maximum bleb wall thickness was significantly thinner after a successful Ahmed valve implantation. They speculated that a thinner bleb allows better aqueous permeability, resulting in better IOP control. However, these authors noted that the wall thickness can change over time. Other studies showed that unlike trabeculectomy, surgeons do not concern with bleb wall properties during tube surgery due to manipulation difficulty, relative homogeneity, and high reflectivity related to fibrous changes in the bleb wall preventing AS-OCT bleb analysis. However, bleb properties during tube surgery are also important for postoperative IOP control [20, 21]. These results were in agreement with another cross-sectional observational study that investigated the role of AS-OCT in the imaging of blebs after AGV surgery in 76 patients. The maximum bleb wall thickness was significantly correlated with the postoperative IOP (*r* = 0.402, *p* < 0.001; *r* = 0.280, *p* = 0.014). AS-OCT clarified that the maximum bleb wall thickness was significantly thinner in successful surgeries when compared to unsuccessful surgeries. No significant differences regarding the bleb wall reflectivity were reported [13].

Resection of the Tenon capsule in AGV surgery was a point of interest in a recent study where 30 patients with refractory glaucoma were randomly divided into 2 groups: group I: AGV surgery with resection of the Tenon capsule and grafting was performed in 15 eyes; group II: AGV surgery with autologous scleral graft was performed in 15 eyes. Better surgical outcomes were observed in group I in terms of the IOP-lowering effect and low number of postoperative antiglaucomatous medications (6.7%), in addition to a low incidence of complications such as one case of hypotony (6.7%) and only one case (6.7%) presenting with the hypertensive phase, which has been explained in this study as a consequence of the low incidence of fibrosis that might occur after resection of the Tenon capsule [7].

Other studies showed the significance of changes in bleb wall morphology, as one study enrolled 52 patients who underwent AGV implantation. Postoperative IOP was decreased at each time point during the postoperative follow-up (*p* value <0.001, all), with a peak in the first month. Mean bleb wall thickness was the thinnest 1 month after the surgery. A hypertensive phase was reported in 44 patients (84.6%). This study was different regarding the bleb wall reflectivity, where there was a significant difference between the hypertensive group and the nonhypertensive group at 1 month postoperatively (130.67 ± 27.00 versus 106.57 ± 10.35; *p* = 0.044) [22].

A study that compared the conventional AGV surgery method and the Biodegradable Collagen Matrix–Augmented Ahmed Glaucoma Valve method included 43 refractory glaucoma eyes that were followed up for 6 months. The conventional method was performed in 21 eyes, and the Biodegradable Collagen Matrix–Augmented Ahmed Glaucoma Valve method was performed in 22 eyes. Maximal bleb thickness was measured using AS-OCT images, and postoperative blebs were imaged using a Spectralis OCT (Heidelberg Engineering GmbH, Heidelberg, Germany) on postoperative days 1, 30, and 180. The results revealed that the Biodegradable Collagen Matrix–Augmented Ahmed Glaucoma Valve method provided better surgical outcomes and a more maximal bleb wall thickness at 1 and 3 months and, therefore, a less hypertensive phase compared to the conventional AGV surgery method. [23].

### 5.1. Strengths and Limitations

The limitations of this study were a relatively small number of patients and a short-term follow-up. In addition, bleb imaging using AS-OCT required expertise to analyse the measures, and sometimes it was difficult to obtain good results, especially with uncooperative patients. In the near future, we plan to work on a large sample with a long-term follow-up to detect the degree of effectiveness of this surgical technique and to evaluate the long-term changes in the bleb. Strengths of this study were the effectiveness of the AGV surgery with Tenon resection, low cost, minimal time consumption, and an easy dissection in the absence of cicatrising tissues from previous surgeries. AS-OCT was added as a point of interest in this study and may provide important diagnostic data to the previous study that we published, wherein we had clarified the role of Tenon resection in reducing postoperative fibrosis, and therefore the hypertensive phase. AS-OCT was useful to evaluate the changes in bleb morphology during the early postoperative period, when the hypertensive phase may occur at 1–3 months. AS-OCT was also useful to perform a correlation between the bleb morphological changes and the bleb function and, therefore, helped to evaluate the success of the surgery.

## 6. Conclusions

AS‐OCT can be used as a good diagnostic tool for analysing the bleb shape, wall thickness, and wall reflectivity, as well as a prognostic tool that determines the incidence of the hypertensive phase. There was a significant increase in bleb wall thickness in AGV surgery with resection of the Tenon capsule at 1 and 3 months compared to the conventional AGV surgery, which may be the cause for the lower hypertensive phases that occurred in the AGV surgery with resection of the Tenon capsule.

## Figures and Tables

**Table 1:** Demographic data and baseline clinical data.

	Group 1		Group 2		*p* value
	No = 15		No = 15		
	NO	%	NO	%	
Age (years)	46.33 ± 1.47		47.14 ± 3.33		0.399
Gender					
Male	8	53.3	9	60.0	0.713
Female	7	46.7	6	40.0	
Preoperative baseline IOP (mean ± SD)	32.73 ± 2.12		33.2 ± 2.21		0.560
Type of glaucoma					
Aphakic glaucoma	5	33.4	6	40.0	0.924
Pseudophakic glaucoma	8	53.3	7	46.7	
Psedoexfoliation glaucoma	2	13.3	2	13.3	
Preoperative medications					
Topical(beta-blocker + CAI + prostaglandin analogue)	13	86.7	12	80.0	0.624
Topical (beta-blocker + CAI + brimonidine)	2	13.3	3	20.0	

IOP = intraocular pressure; SD = standard deviation; CAI = carbonic anhydrase inhibitor.

**Table 2:** IOP of the studied groups.

IOP (mmHg)	Group 1	Group 2	*p* value
	No = 15	No = 15	
Preoperative			
Mean ± SD	32.73 ± 2.12	33.2 ± 2.21	0.560
Postoperative 1 day			
Mean ± SD	11.4 ± 2.29	12.33 ± 3.68	0.202
Postoperative 1 week			
Mean ± SD	12.07 ± 1.53	13.27 ± 3.03	0.098
Postoperative 1 month			
Mean ± SD	14.87 ± 3.11	20.13 ± 6.39	**0.016** ^**∗**^
Postoperative 3 months			
Mean ± SD	15.8 ± 3.41	17 ± 1.81	**0.01** ^**∗**^
Postoperative 6 months			
Mean ± SD	13.33 ± 1.59	14.27 ± 1.44	0.103
Fried man test (*p* value)	77.69 **(<0.001**^**∗**^**)**	80.04 **(<0.001**^**∗**^**)**	
Post hoc test	^**1,2,3.4.5.7,8,10,11**^ **<0.05**	^**1,2,3.4.5.7,8,10,11,14,15**^ **<0.05**	
	^**6,9,12,13,14,15**^ ** >0.05**	^**6,9,12,13**^ ** >0.05**	

^1^Preoperative-1 day later; ^2^preoperative-1 week later; ^3^preoperative-1 month later; ^4^preoperative-3 months later; ^5^preoperative-6 months later; ^6^1 day later-1 week later; ^7^1 day later-1 month later; ^8^1 day later-3 months later; ^9^1 day later-6 months later; ^10^1 week later-1 month later; ^11^1 week-3 months later; ^12^1 week-6 months later; ^13^1 month later-3 months later; ^14^1 month-6 months later; ^15^3 months-6 months later. ^*∗*^Significant.

**Table 3:** Postoperative complications of the studied groups.

Postoperative complications	Group I		Group II		*p* value
	No = 15		No = 15		
	NO	%	NO	%	
Hypotony					
No	14	93.3	12	80.0	0.598
Yes	1	6.7	3	20.0	
Shallow AC					
No	14	93.3	12	80.0	0.598
Yes	1	6.7	3	20.0	
Increased IOP					
Negative	13	86.7	9	60.0	0.215
Positive	2	13.3	6	40.0	
Tube exposure					
Negative	14	93.3	13	86.7	0.483
Positive	1	6.7	2	13.3	
Choroidal detachment					
Negative	15	100.0	14	93.3	0.734
Positive	0	0.0	1	6.7	
Hyphema					
Negative	14	93.3	13	86.7	0.974
Positive	1	6.7	2	13.3	
Continuity of antiglaucoma medications					
Two medications	1	6.7	3	20.0	0.513
Three medications	0	0.0	1	6.7	
No medications	14	93.3	11	73.3	

^*∗*^Significant.

**Table 4:** Maximal bleb wall thickness of the studied groups.

Postoperative maximal bleb wall thickness (*μ*m)	Group 1	Group 2	*p* value
	No = 15	No = 15	
1 day			
Mean ± SD	754.67 ± 53.93	707.13 ± 31.7	**0.004** ^**∗**^
Median	780	719	
Min-max	650–800	640–740	
1 week			
Mean ± SD	712.33 ± 66.99	599.93 ± 41.82	**<0.001** ^**∗**^
Median	750	590	
Min-max	610–780	550–690	
1 month			
Mean ± SD	684 ± 81.66	499.53 ± 99.1	**<0.001** ^**∗**^
Median	730	535	
Min-max	530–760	370–625	
3 months			
Mean ± SD	671.6 ± 69.48	506 ± 76.91	**<0.001** ^**∗**^
Median	710	515	
Min-max	545–745	412–615	
6 months			
Mean ± SD	718.2 ± 72.4	620.86 ± 82.29	**0.002** ^**∗**^
Median	760	630	
Min-max	560–785	515–725	
Fried man test (*p* value)	45.329 **(<0.001**^**∗**^**)**	54.247 **(<0.001**^**∗**^**)**	
Post hoc test	^**2,3.6.9,10**^ **<0.05**	^**1,2,3.4.5.6,9,10**^ **<0.05**	
	^**1,4,5,7,8**^ ** >0.05**	^**7.8**^ ** >0.05**	

^1^1 day later-1 week later; ^2^1 day later-1 month later; ^3^1 day later-3 months later; ^4^1 day later-6 months later; ^5^1 week-1 month later; ^6^1 week later-3 months later; ^7^1 week-6 months later; ^8^1 month later-3 months later; ^9^1 month later-6 months later; ^10^3 months later-6 months later. ^*∗*^Significant.

**Table 5:** Maximal bleb wall reflectivity of the studied groups.

Postoperative bleb wall reflectivity	Normotensive patients			Hypertensive phase		
	Group I	Group II	*p* value	Group I	Group II	*p* value
	No = 13	No = 9		No = 2	No = 6	
	Mean ± SD	Mean ± SD		Mean ± SD	Mean ± SD	
1 day	79.46 ± 45.13	83.44 ± 42.11	0.834	81.18 ± 33.11	84.33 ± 22.65	0.921
Week 1	90.28 ± 17.38	94.28 ± 15.67	0.581	99.44 ± 15.33	105.45 ± 18.33	0.694
1 month	98.76 ± 13.11	111.55 ± 11.24	**0.029** ^**∗**^	113.48 ± 38.22	127.66 ± 32.11	0.719
3 months	100.43 ± 44.22	115.36 ± 22.16	0.311	106.31 ± 19.56	121.62 ± 18.23	0.508
6 months	97.23 ± 36.63	102.11 ± 20.11	0.693	99.83 ± 19.48	103.65 ± 13.22	0.839

^*∗*^Significant.

**Table 6:** Maximal bleb wall thickness in patients with hypertensive phase.

Postoperative maximal bleb wall thickness (*μ*m)	Hypertensive phase		*p* value
	Group I	Group II	
	No = 2	No = 6	
Postoperative 1 day			
Mean ± SD	770 ± 28.28	718.67 ± 9.16	**0.044** ^**∗**^
Median	770	718.5	
Min-max	750–790	710–735	
Postoperative 1 week			
Mean ± SD	645 ± 49.49	584.17 ± 7.36	**0.044** ^**∗**^
Median	645	582.5	
Min-max	610–680	575–595	
Postoperative 1 month			
Mean ± SD	540 ± 14.14	387.83 ± 11.67	**0.042** ^**∗**^
Median	540	388.5	
Min-max	530–550	370–400	
Postoperative 3 months			
Mean ± SD	567.5 ± 31.82	423.67 ± 9.31	**0.042** ^**∗**^
Median	567.5	425	
Min-max	545–590	412–435	
Postoperative 6 months			
Mean ± SD	590 ± 42.43	529.83 ± 13.12	**0.044** ^**∗**^
Median	590	527	
Min-max	560–620	515–550	

^*∗*^Significant.

**Table 7:** Correlation between maximal bleb wall thickness and intraocular pressure in studied groups.

Postoperative	Group I		Group II		Total sample	
	*r*	*p*	*r*	*p*	*r*	*p*
1 day	−0.274	0.323	−0.016	0.954	−0.190	0.314
1 week	−0.463	0.082	0.202	0.470	−0.295	0.113
1 month	−0.227	0.416	−0.853	**<0.001** ^**∗**^	−0.702	**<0.001** ^**∗**^
3 months	−0.275	0.322	−0.491	0.063	−0.611	**<0.001** ^**∗**^
6 months	−0.228	0.414	−0.689	**0.004** ^**∗**^	−0.512	**<0.001** ^**∗**^

^*∗*^Significant.

**Table 8:** Correlation between maximal bleb wall thickness and maximal bleb wall reflectivity in studied groups.

Postoperative follow-up period	Group I		Group II		Total sample	
	*r*	*p*	*r*	*p*	*r*	*p*
1 day	−0.197	0.323	−0.113	0.209	−0.168	0.239
1 week	−0.385	0.082	−0.109	0.145	−0.295	0.096
1 month	−0.486	**0.032** ^**∗**^	−0.327	**0.019** ^**∗**^	−0.409	**0.022** ^**∗**^
3 months	−0.609	**0.002** ^**∗**^	−0.491	**0.005** ^**∗**^	−0.589	**0.003** ^**∗**^
6 months	−0.535	**0.008** ^**∗**^	−0.432	**0.009** ^**∗**^	−0.495	**0.007** ^**∗**^

^*∗*^Significant.

## Data Availability

The data used to support the findings of this study are included within the article.
